# CD47-SIRPα Checkpoint Disruption in Metastases Requires Tumor-Targeting Antibody for Molecular and Engineered Macrophage Therapies

**DOI:** 10.3390/cancers14081930

**Published:** 2022-04-11

**Authors:** Jason C. Andrechak, Lawrence J. Dooling, Michael P. Tobin, William Zhang, Brandon H. Hayes, Justine Y. Lee, Xiaoling Jin, Jerome Irianto, Dennis E. Discher

**Affiliations:** 1 Biophysical Engineering Labs, University of Pennsylvania, Philadelphia, PA 19104, USA; jandrech@seas.upenn.edu (J.C.A.); ldooling@seas.upenn.edu (L.J.D.); mptobin9@seas.upenn.edu (M.P.T.); zhangwil@seas.upenn.edu (W.Z.); hbrandon@seas.upenn.edu (B.H.H.); jyl53@seas.upenn.edu (J.Y.L.); xiaoling.jin@pennmedicine.upenn.edu (X.J.); jerome.irianto@med.fsu.edu (J.I.); 2 Graduate Group of Bioengineering, University of Pennsylvania, Philadelphia, PA 19104, USA; 3 Department of Biomedical Sciences, College of Medicine, Florida State University, Tallahassee, FL 32306, USA

**Keywords:** macrophage checkpoint, CD47, metastasis, melanoma, cell therapy, SIRPα, B16F10, immunocompetent, immunotherapy

## Abstract

**Simple Summary:**

Immunotherapies offer broad potential to treat many diseases, including cancer. Macrophages are highly abundant immune cells, but are generally inhibited from eliminating tumor cells by ‘self’ signaling. Blocking this macrophage signaling checkpoint with antibodies has gone through clinical trials, which show that efficacy remains elusive especially for solid tumors. Here we show that CRISPR-based deletion of this inhibitory checkpoint is also insufficient to suppress solid metastatic tumors unless combined with a pro-phagocytic tumor-opsonizing antibody. Our tumor model is immunocompetent and resistant to T cell checkpoint blockade, which are typical characteristics of human cancer. We further demonstrate an equally effective cell therapy with antibody-engineered macrophages, and the approach also suppresses model metastatic human tumors.

**Abstract:**

The macrophage checkpoint interaction CD47-SIRPα is an emerging target for cancer therapy, but clinical trials of monoclonal anti-CD47 show efficacy only in liquid tumors when combined with tumor-opsonizing IgG. Here, in challenging metastatic solid tumors, CD47 deletion shows no effect on tumor growth unless combined with otherwise ineffective tumor-opsonization, and we likewise show wild-type metastases are suppressed by SIRPα-blocked macrophages plus tumor-opsonization. Lung tumor nodules of syngeneic B16F10 melanoma cells with CD47 deletion show opsonization drives macrophage phagocytosis of B16F10s, consistent with growth versus phagocytosis calculus for exponential suppression of cancer. Wild-type CD47 levels on metastases in lungs of immunocompetent mice and on human metastases in livers of immunodeficient mice show that systemic injection of antibody-engineered macrophages also suppresses growth. Such in vivo functionality can be modulated by particle pre-loading of the macrophages. Thus, even though CD47-SIRPα disruption and tumor-opsonizing IgG are separately ineffective against established metastatic solid tumors, their combination in molecular and cellular therapies prolongs survival.

## 1. Introduction

The CD47-SIRPα ligand-receptor interaction between myeloid and ‘self’ cells serves as a ubiquitous immune checkpoint to regulate the phagocytic clearance of ‘foreign’ pathogens, materials, and debris relative to healthy ‘self’ cells [1]. CD47 signals “don’t eat me” to macrophages by passivating phagocytic activation of the cytoskeleton [2]. Recent interest in CD47 as a cancer therapeutic target has led to rapid expansion of preclinical and clinical efforts to physically block its inhibitory signaling in liquid and solid tumors that express CD47 at normal or elevated levels [3,4]. CD47-blocking antibodies in human trials have demonstrated promising early results in lymphoma that indicate a requirement for combination with a tumor-opsonizing IgG (e.g., anti-CD20, rituximab) [5]. However, efficacy with anti-CD47 antibodies in solid tumors remains unclear in the clinic [4], and a pre-clinical replication study of a syngeneic immunocompetent mouse model also cast doubt on early reports of success in mouse models with anti-CD47 monotherapy [6]. More generally, some immunotherapies (e.g., engineered T cells) show impressive success against liquid tumors with durable long-term responses [7,8,9], but solid tumors remain a general challenge—especially with poorly immunogenic tumors and with metastatic tumors that are most relevant to patients. 

**Figure 1:**
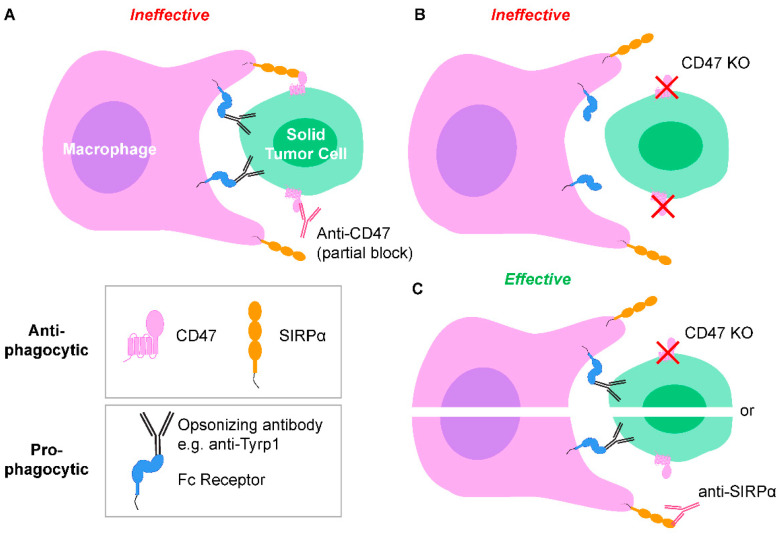
CD47-SIRPα signaling and pro-phagocytic cues between macrophages and solid tumor cells. (**A**) Fc regions of tumor-opsonizing antibodies bind to Fc receptors on macrophages, and Fab regions bind to tumor cell antigens. These signals act as a pro-phagocytic cues, but phagocytosis is ineffective when CD47 is only partially blocked by anti-CD47. (**B**) Complete knockout (KO) of tumor cell CD47 to ablate CD47-SIRPα signaling without a pro-phagocytic signal results in ineffective phagocytosis. (**C**) Both tumor cell CD47 KO (or anti-SIRPα block on macrophages) and tumor-opsonizing antibodies results in effective phagocytosis.

**Figure 2:**
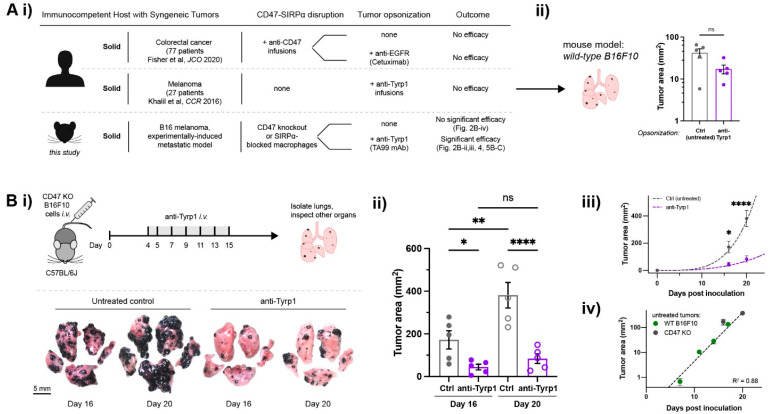
Tumor-opsonizing antibodies can drive regression of metastases only when CD47 is disrupted. (**A**) (i) Human clinical CD47 blockade in solid tumors compared to our main design of a suitable preclinical model (mouse). Anti-CD47 monotherapies show no efficacy, motivating a combination with tumor-opsonizing antibodies (e.g., Cetuximab in colorectal cancer). Anti-Tyrp1 monotherapy also showed no efficacy against human melanoma. Two distinct approaches are taken in our model. (ii) Anti-Tyrp1 opsonization of established wild-type (WT) B16F10 melanoma metastases does not significantly affect lung tumor burden on day 14 after tail-vein inoculation with 2 × 10^5^ B16F10 cells. Tumors are allowed to establish, and mice are then tail-vein injected with anti-Tyrp1 (250 µg dosing) on days 4, 5, 7, 9, 11, and 13. Mice are sacrificed and organs are surveyed for B16F10 metastases. Results are illustrative of three independent experiments (*n* = 5 per group, mean ± s.e.m., n.s. not significant for *p* > 0.05, unpaired t-test). (**B**) CD47-deleted lung metastases require anti-Tyrp1 for significant suppression. (i) Mice are tail-vein injected with 2 × 10^5^ CD47 KO B16F10 cells on day 0 and later treated by tail-vein injections of anti-Tyrp1 (75 µg dosing). Mice are sacrificed and organs are surveyed for metastases, but only the lung shows nodules. Representative images from untreated controls or anti-Tyrp1 treatment. (ii) Tumor area on both sides of lung lobes were quantified, showing significant suppression by anti-Tyrp1 (*n* = 5 per group, mean ± s.e.m.; * *p* < 0.05, ** *p* < 0.01, **** *p* < 0.0001, one-way ANOVA, uncorrected Fisher’s LSD). (iii) Exponential-type growth (as power law: Area = A_0_ t^B^: A_0_,_ctrl_ = 0.0019, A_0_,_tx_ = 0.0087, B_ctrl_ = 4.1, B_tx_ = 3.0). (iv) CD47 deletion does not affect tumor growth relative to WT (Area = A_0_ e^k*t^, where A_0_ = 0.05, k = 0.45).

Infusions of species-relevant anti-CD47 IgG (e.g., mouse IgG into mouse, or humanized IgG into human) cause loss of blood cells, which confirms biological activity but raises concerns over safety [5,6]. Such on-target, off-tumor effects underscore the limited efficacy of monotherapy and motivate an increasing focus on combination therapies (Figure 1 and Figure 2Ai) [3,4,5,10,11,12,13,14]. Trials are now beginning with anti-SIRPα, but SIRPα is expressed on many cell types and clinical advantages remain unclear [15,16,17]. Transport of antibodies and effector cells into solid tumors including metastatic lesions can also be problematic.

Here, we focus first on poorly immunogenic, syngeneic B16F10 melanoma cells in lung [18] to model solid tumor metastasis within immunocompetent C57BL/6 mice. Deletion of CD47 on opsonized B16F10s increases the engulfment of cell suspensions in vitro by bone marrow-derived macrophages in our recent studies [19]. We now find that in vivo efficacy against established B16F10 metastatic nodules requires both CD47 deletion and a pro-phagocytic, melanocyte-specific IgG. Our quantitative analyses of tumor growth with CD47 deletion together with high-resolution imaging indicates on-target phagocytosis with IgG opsonization in B16F10 tumor suppression. In a second approach, we modify marrow macrophages for an adoptive cell therapy by pre-blocking SIRPα and pre-loading Fc-receptors with tumor-opsonizing IgG prior to infusion. Human lung carcinoma A549 liver metastases in immunodeficient mice confirm the effects of this cell therapy and thus confirm the benefits of the combination approach.

## 2. Results

### 2.1. Tumor-Opsonizing Antibodies Drive Regression of Metastases Only When CD47 Is Disrupted

We used CRISPR/Cas9 to eliminate CD47 from the surface of B16F10 cells without the affecting surface levels of another relevant protein, tyrosinase-related protein 1 (Tyrp1; Figure S1) [19]. Tyrp1 is targetable in vivo with a mouse monoclonal IgG2a antibody (anti-Tyrp1 clone TA99) that activates Fc receptors (FcR) [20,21] and can suppress wild-type (WT) B16F10 tumors if injected within ~1 day of B16F10 inoculation [20,22]. TA99 monotherapy is generally ineffective in established WT B16F10 tumors (Figure 2Aii and Figure S2A,B), which concurs with recent clinical trial results of anti-TYRP1 monotherapy against human melanoma that demonstrated safety but no anti-tumor efficacy [23]. Our dosage range for TA99 is similar to that in the clinical trial: 1–10 mg/kg was here compared to safety dose testing of 5, 10, 20, and 30 mg/kg in the trial.

For all in vivo B16F10 experiments, metastases are allowed to establish in the lungs prior to tail-vein injections of anti-Tyrp1 (Figure 2Bi: treatment from days 4 to 15). Tumor burden was quantified from melanized area and/or nodules on the surfaces of mouse lung lobes post-sacrifice (Figure S2C,D); we avoid live-animal imaging methods such as those using luciferase because they add immunogenicity. For WT B16F10 metastases expressing wild-type levels of CD47, high TA99 doses (250 µg: ~10 mg/kg) show no significant effect on lung tumors compared to untreated or isotype IgG2a-treated controls 14 days after tumor inoculation (Figure 2Aii and Figure S2B). Yet, when CD47 is deleted from the tumors, even moderate doses (75 µg, ~3 mg/kg) suppress growth even at days 16 and 20 (Figure 2Bi–iii). Whereas untreated lungs show melanoma covering nearly the entire lung surface, anti-Tyrp1-treated lung lobes exhibit relatively few, smaller nodules.

We show that the anti-Tyrp1 doses are effective against established CD47 KO tumors, whereas the WT tumors show no significant change (Figure S2A), even when systemic injections of murine anti-CD47 are combined with the anti-Tyrp1 (Figure S3). The latter result might be explained in part by previous observations that systemic anti-CD47 can only access and bind a fraction of CD47 on the surface of these tumor cells [24,25]. Thus, in contrast to WT tumors, complete disruption of CD47′s inhibitory “don’t eat me” signal enables FcR-mediated phagocytosis to suppress growth of CD47 KO metastatic tumors. Most past studies of WT that show efficacy of TA99 began treatment on days 0 to 1 after tumor inoculation [22,24,25], affecting initial cell engraftment rather than treating the more clinically relevant, established tumors. Even in one early study where TA99 was injected later [22], mAb doses were ~8-fold higher than here.

CD47-deleted lung metastases across a cohort measured at different time points exhibit exponential (or power law) growth, and the growth is identical to WT tumors (Figure 2Biii,iv). These results align with current clinical results that generally show that anti-CD47 monotherapy does not affect tumor growth. Our studies show that tumor variance increases with time, which is consistent with the exponential-type growth that is typical of unhindered proliferation, including B16 tumors at subcutaneous sites [26]. Longitudinal measurements of B16 lung metastasis growth are challenging without modified cells (e.g., without immunogenic reporters), but one study that showed similar exponential-type B16 growth used computed tomography (CT) [27]. Our own CT imaging of tumor margins (Figure S4) suggested higher resolution and higher confidence in the mean kinetics when measured by optical imaging of a cohort at sacrifice (Figure S2). Overall, the doubling of CD47 KO metastases is suppressed ~4–5-fold by anti-Tyrp1 (Figure 2Biii), suggesting that anti-Tyrp1 drives tumor cell phagocytosis.

### 2.2. Macrophages Infiltrate and Phagocytose CD47-Depleted, IgG-Opsonized B16F10 Nodules

**Figure 3:**
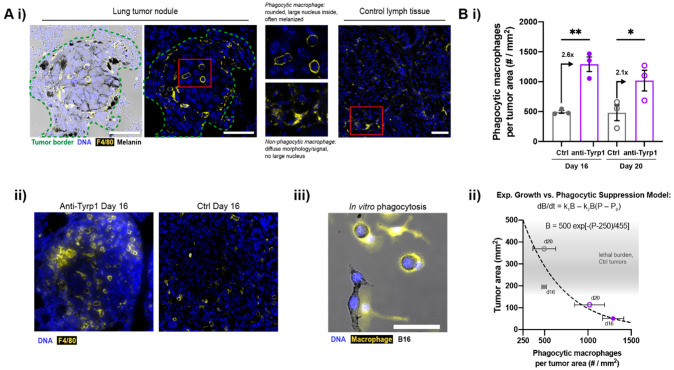
Tumor-opsonizing antibodies increase macrophage infiltration in tumors. (**A**) Phagocytic macrophages in treated CD47 KO lung tumor nodules. (i) Confocal images of anti-Tyrp1-treated CD47 KO tumors show F4/80+ macrophages that are rounded in morphology and contain melanin and large DAPI+ nuclei, indicating phagocytosis of B16F10 cells. F4/80+ macrophages in control lymph tissue (lymphatics-localized) show diffuse, non-phagocytic morphology. (ii) Treated lung nodules show more phagocytic macrophages than untreated control lungs. (iii) Rounded macrophage in vitro after engulfing an opsonized B16F10 cell. Scale: 50 µm. (**B**) (i) Phagocytic macrophages per tumor area increase with anti-Tyrp1 treatment in CD47 KO lung tumor nodules. Mean ± s.e.m. (* *p* < 0.05, ** *p* < 0.01, Student’s *t*-test, *n* = 3 representative samples per group, closed circles indicate day 16 samples and open circles indicate day 20 samples). (ii) Relationship between the lung tumor area and density of phagocytic macrophages: exponential-type growth versus phagocytic suppression. Tumor burden, B, is modelled by an ordinary differential equation in which growth is suppressed by phagocytic interactors, P. P_0_ refers to inactivated macrophages.

To assess phagocytosis in the melanized tumor nodules, late-stage lung sections were immunostained for macrophage marker F4/80 (Figure S5). We also noted that the DAPI-stained nuclei in nodules are large and consistent with B16′s high-modal karyotype [28]. Importantly, CD47 KO tumor nodules treated with anti-Tyrp1 show many F4/80-positive cells that are round in morphology and contain a single large nucleus plus some melanization (Figure 3Ai,ii). Such features indicate in vivo phagocytosis of a B16F10 cell. Lymphatics-localized F4/80+ macrophages are distinct and always appear more spread. Bone marrow-derived macrophages in culture are similarly spread on plastic unless they have phagocytosed an anti-Tyrp1-opsonized B16F10 cell, in which case they are round and melanized (Figure 3Aiii). Increased tumor infiltration by immune cells is further suggested by a marker-free analysis of nuclear size and intensity (small and bright) (Figure S6).

Untreated tumor nodules show the same low density of phagocytic F4/80+ macrophages at days 16 and 20, and 2.6-fold more on day 16, with 2.1-fold more on day 20 (Figure 3Bi). The modest decrease is consistent, with rapid B16 proliferation and tumor growth (Figure 2Biii) progressively dominating the clearance by these effector immune cells. We model the net growth rate of metastatic tumors as an exponential growth solution to a differential equation where the tumor growth rate increases with tumor cell proliferation and decreases as a function of macrophage phagocytosis (Figure 3Bii, equations). This exponential fits well with the total tumor burden versus the number of phagocytic macrophages (Figure 3Bii, plot), and illustrates the disproportionate potency of tumor clearance possible when CD47 disruption is combined with IgG opsonization.

### 2.3. Tumor Clearance Induced by Opsonization and CD47 Deletion Prolongs Survival, but Some Tumors Escape

**Figure 4:**
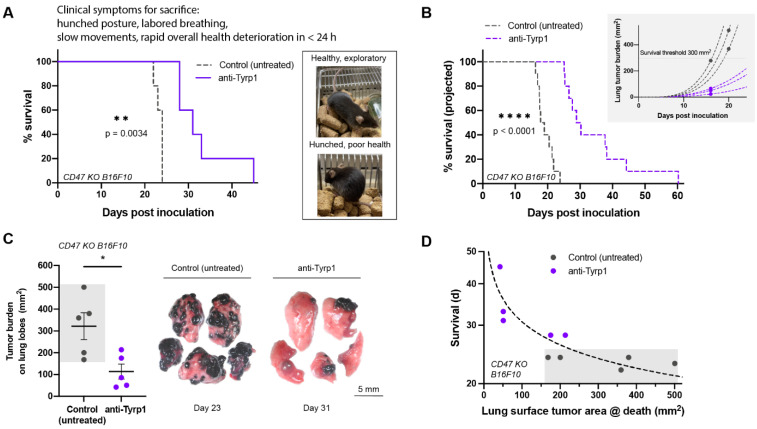
Tumor clearance induced by opsonization AND CD47 deletion prolongs survival, but a few large tumor masses still cause death. (**A**) Survival of anti-Tyrp1-treated mice induced with 2 × 10^5^ CD47 KO B16F10 cells intravenously. 250 µg anti-Tyrp1 doses beginning on Day 4 post inoculation prolong the survival of mice by about 30%, but mice are unable to be cured completely (*n* = 5 per group, ** *p* < 0.01, Log-rank test). Right: mice are healthy and exploratory, then rapidly deteriorate in health in less than 24 h, showing clinical symptoms of hunched posture, labored breathing, slow reactivity, and weight loss. (**B**) Projected survival of anti-Tyrp1-treated mice induced with 2 × 10^5^ CD47 KO B16F10 intravenously modeled with a power law fit based on single timepoint estimates of growth. With 75 µg of anti-Tyrp1 on the same dosing timeline, the estimated projected survival advantage is ~6–7 days (*n* = 10 per group, **** *p* < 0.0001, Log-rank test). Inset: exponential-type growth projections of six representative mice. (**C**) CD47 KO B16F10 tumor burden on lung lobes at sacrifice. Anti-Tyrp1-treated mice have lungs with markedly reduced tumor burden relative to untreated mice, yet demonstrate similar clinical symptoms, requiring sacrifice (*n* = 5 per group, * *p* < 0.05, unpaired t-test). (**D**) Survival of mice in days versus lung surface tumor area at sacrifice. An exponential-type model shows that survival is inhibited by lung tumor burden.

To directly assess survival, we followed small cohorts of untreated and treated mice over time. All untreated mice with CD47 KO lung metastases exhibited numerous clinical symptoms that required euthanasia (hunched posture, slow movement, labored breathing), whereas mice treated with anti-Tyrp1 showed a median survival ~30% longer (Figure 4A). Furthermore, untreated controls all required sacrifice within a narrow window of 2–3 days (at ~23 days), whereas all mice in the treatment group survived longer, with a large variation and 20% surviving up to 2-fold longer.

Applying our nonlinear analyses (per Figure 2B and Figure 3B) to single snapshot measurements on sacrificed mice (e.g., day 16), we estimated survival curves of cohorts (Figure 4B) that align very well with our direct measurements of survival (Figure 4A). More specifically, we fit an exponential-type curve to the datapoint for each sacrificed mouse, and then we identify when the curve crosses a critical tumor burden (see Methods). The Kaplan–Meier curves show that the projected long-term survival is nearly the same as the measured survival, with 20% again living twice as long or longer than the controls (~20 days). Although these experiments used different anti-Tyrp1 doses in the treated cohorts (3 mg/kg vs. 10 mg/kg), the overall agreement suggests that the higher anti-Tyrp1 dose in the symptoms-based cohort is in therapeutic excess.

Strikingly, despite the similar clinical symptoms between treated and untreated mice, lower lung tumor burden was present on the long-surviving, treated mice (Figure 4C,D). Mice with the lowest lung tumor burden measured at sacrifice survived the longest. Clinical symptoms leading to rapid decompensation were consistent with compression of airways, blood vessels, and surrounding lung tissues leading to hypoxia, and post-mortem dissection indeed showed a few large tumor masses (Figure S7). We confirmed the identity of these melanized masses as B16F10 tumors by disaggregating and growing the cells in culture. Thus, a small number of metastases can outpace clearance by macrophages and cause death in this fast-growing model. Regardless, complete CD47 disruption combined with a tumor-opsonizing antibody significantly suppresses metastatic-type tumor growth and prolongs survival.

### 2.4. Antibody-Engineered Marrow Macrophages/Monocytes Eliminate Lung Tumor Nodules in WT Metastases, While Anti-Tyrp1 Treatment Alone Does Not

**Figure 5:**
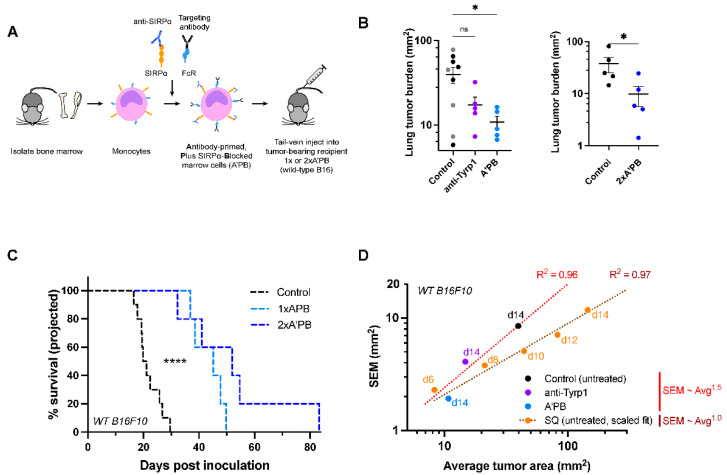
SIRPα-blocked, Tyrp1-targeted marrow cells suppress wild-type metastases, while anti-Tyrp1 treatment alone does not. (**A**) Schematic of SIRPα-blocked, Tyrp1-targeted (A’PB) fresh marrow cell production for tail-vein infusion into mice with WT B16F10 lung metastases. Monocytes and macrophage pre-cursors are the main marrow cells with SIRPα and Fc receptors, and the latter are pre-loaded with anti-Tyrp1 prior to infusion. Mice receive 2 × 10^7^ antibody-engineered marrow cells intravenously on day 4 (1× A’PB) and 6 (2× A’PB). Additional anti-Tyrp1 was administered i.v. on days 4, 5, 7, 9, 11, and 13 (250 µg doses). (**B**) WT B16F10 tumor burden in mice treated with antibody-engineered marrow. Left: Control mice were treated with either isotype IgG2a antibody (gray, 250 µg dosing regimen) or received no treatment (black), while treated mice received either anti-Tyrp1 (250 µg dosing regimen) or A’PB marrow with anti-Tyrp1 (250 µg dosing regimen). Mean and s.e.m. (* *p* < 0.05, n.s. not significant, ordinary one-way ANOVA, *n* = 5 per group except isotype control *n* = 4). Right: Tumor burden in a separate experiment comparing control (untreated) or 2× A’PB groups. Lung tumor burden was assessed on day 14 post-inoculation. Mean and s.e.m. (* *p* < 0.05; this data is part of the experiment in Figure 7B, which describes the statistical test). (**C**) Projected survival advantages in A’PB-treated lung tumors. (n = 10 for control group, n = 5 per treatment group, **** < 0.0001, Log-rank test). (**D**) Variance of tumor areas scales with average tumor area of lung nodules, consistent with power law growth.

WT metastases that express CD47 are more clinically relevant than CD47 KO tumors, and so we sought a translatable approach to the treatment of WT B16F10 lung metastases. To do this, we extended an adoptive cell therapy we had developed [29], in which an anti-SIRPα blocking antibody is added to fresh marrow macrophages and monocytes together with anti-Tyrp1 loaded onto Fc receptors (Figure 5A). Tail-vein injections of these Antibody-primed Plus SIRPα-Blocked (A’PB) engineered macrophages previously proved to be safe and effective against subcutaneous human tumor lines in immunodeficient mice but had not been tested on metastases in syngeneic, immunocompetent models. Our earlier studies also showed that sorting of macrophages/monocytes from marrow gave the same results in terms of tumor suppression as unsorted marrow, likely because those cell fractions are the main marrow cell types that express SIRPα. Pre-incubation of freshly isolated marrow maximizes the saturable antibody binding to the effector cells and distinguishes our antibody-engineered macrophage approach from systemic injections of antibodies. In particular, systemic anti-SIRPα will preferentially bind circulation-accessible SIRPα, such as the SIRPα displayed on splenic macrophages and on liver Kupffer macrophages [30].

A’PB cells were tail-vein injected into mice with established lung metastases (2 × 10^7^ A’PB at day 4 post-inoculation). To continue to drive phagocytosis via tumor opsonization, anti-Tyrp1 was administered per above (Figure 2Bi). WT metastases are clearly suppressed by A’PB (Figure 5B and Figure S8), whereas anti-Tyrp1 does not show significant effects relative to controls including isotype IgG2a (in agreement with Figure 2Aii and Figure S2A,B). Survival estimations for these day 14 timepoints show prolonged survival with A’PB, giving a projected median survival that is almost 2-fold longer than the control cohort (again showing ~23 day median survival) (Figure 5C).

To further enhance the anti-tumor efficacy, a second dose of A’PB at day 6 (denoted as 2× A’PB) was administered, which confirmed significant suppression of tumor growth (Figure 5B) and prolonged the projected survival. Compared to the median survival of the control cohort in this separate study (~25 days), all of the treated cohort survived longer (Figure 5C), and 20% were projected to survive 3–4-fold longer (82 days), which is the longest survival time in any of our studies. Multiple doses of our antibody-engineered marrow macrophage/monocyte therapy can thus offer further opportunity to enhance suppression of WT metastases.

Mathematically, any given cohort within an experiment shows higher variation for larger mean tumor size (Figure 5D), which is expected for any set of exponential-type growth curves that start with small engraftment differences (e.g., Figure 2Biii). Moreover, the mouse-to-mouse variation (s.e.m.) in the sizes of lung metastases scales with mean for a cohort, with similar findings for subcutaneous tumors. Greater variation with lung metastases seems consistent nonetheless with post-intravenous engraftment of multiple nodules (Figure 2Bi). These trends in variance allow us to use our exponential-type fits of day 14 data (Figure 2B) and calculate both the mean size and s.e.m. for days 16 and 20, with our best estimates showing WT versus (WT + anti-Tyrp1) do not differ significantly. Such modeling can thus minimize unnecessary animal testing, particularly given the very good agreement in measured versus projected survival (Figure 4A,B). Overall, our results for WT CD47 metastases agree with the CD47 deletion results and suggest a promising therapeutic strategy to suppress tough-to-treat metastases.

### 2.5. WT Human Lung Cancer Metastases in Liver Suppressed by Antibody-Engineered Marrow Macrophages/Monocytes

**Figure 6:**
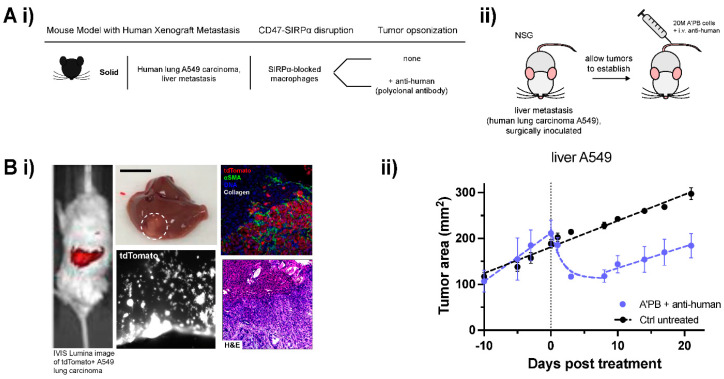
A’PB therapy represses growth in human metastatic WT CD47 tumors. (**A**) (i) Table of human xenograft mouse model details. Human lung A549 carcinoma is surgically implanted at an orthotopic metastatic site in the livers of NSG mice. Anti-SIRPα is used to block CD47 signaling on adoptively transferred marrow cells and anti-human is the source of tumor-opsonizing antibody (A’PB). (ii) Schematic of tumor induction and treatment. NSG mice were surgically implanted with liver metastases. Mice were treated with A’PB marrow on the indicated day 0, and 3 times weekly for 2 weeks with anti-human i.v. (**B**) (i) Representative whole-body fluorescence image of tdTomato signal in surgically implanted liver metastases (left). Images of explanted liver with liver metastasis (middle top, A549 tumor circled, scale: 1 cm) and tdTomato signal of A549s infiltrating stroma (middle bottom). (ii) Sectioned liver tissue images showing tdTomato, αSMA, DNA, and collagen of A549 metastases relative to normal liver tissue (top right) and H&E staining (bottom right). (ii) In vivo growth curve of liver metastatic human lung adenocarcinoma A549 tumors. A’PB and tumor-opsonizing antibody repress tumor growth by 50% over several days relative to untreated control. Tumor burden is monitored by fluorescent imaging with an IVIS Lumina II (fluorophore: tdTomato). Without additional treatment to continue tumor cell clearance, metastases begin to expand again after about 10 days. Error bars represent s.e.m. Exponential decay for Tumor area in the regression phase of the treated tumor fits to exp(−t/1.55) for 0 < t < 8.

To assess the A’PB antibody-engineered marrow macrophages/monocytes in a second metastatic model and to assess relevance to humans, liver metastases of human lung cancer A549 tumors were first established in immunocompromised NSG mice (Figure 6A,B), as previously described by our group [31]. A549 expression of tdTomato enables imaging of infiltrative nodules after engraftment in liver via surgical implantation (Figure 6Bi,ii). Macrophages in NSG mice express a SIRPα variant that favors engraftment of human cells due to its high affinity with human CD47, but NSG mice lack antibodies and functional T cells, B cells, and NK cells [32,33]. Compared to B16F10 tumors that show exponential phase growth from 100 to 200 mm [2] in 2–3 days (Figure 2Biv), A549 tumors grow more slowly with linear growth to the same extent in ~2 weeks (Figure 6Biii).

A’PB treatment of these liver metastatic A549 tumors combined with a tumor-opsonizing antibody regimen (polyclonal anti-human IgG) led to exponential tumor shrinkage of ~50% within days, consistent with our model for growth vs. phagocytic clearance (Figure 6Bii). Regression is transient, but aligns with our previous results for A549 subcutaneous tumors that had shown (i) that an opsonizing antibody alone has no significant effect and that (ii) injected macrophages show progressively less evidence of phagocytosis over time as they differentiate toward a tumor-associated macrophage phenotype [29]. These results further illustrate that for slow-growing solid tumors, macrophage phagocytosis can be engineered or otherwise controlled to dominate tumor growth, in agreement with the proposed mathematical model (Figure 3Bii). B16F10 melanoma tumors grow so rapidly that phagocytosis can only slow the tumor doubling time, but human melanomas grow slowly at rates of <1 mm per month [34]. A549 tumors grow significantly faster at >10 mm per month. Hence, antibody-engineered marrow macrophages/monocytes in marrow with SIRPα-blockade and clinically relevant anti-Tyrp1 [23] can in principle out-compete and thereby repress human metastatic melanoma.

### 2.6. Antibody-Engineered Marrow Macrophages/Monocytes Are Less Effective When Pre-Loaded with Microparticles

**Figure 7:**
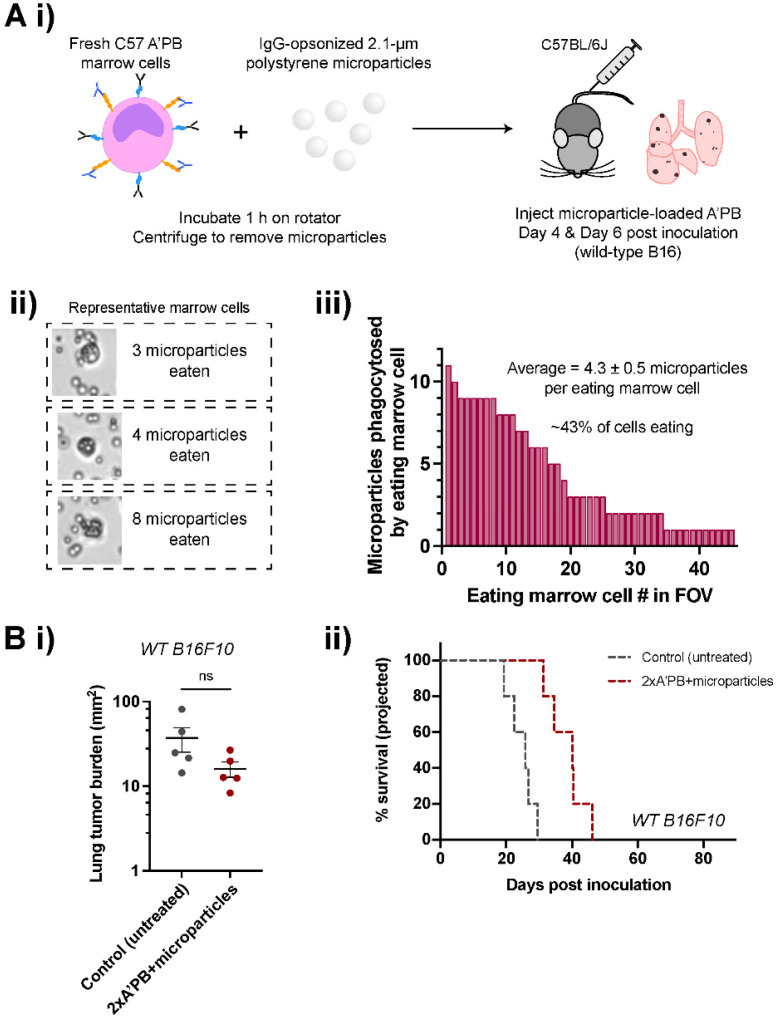
Pre-loading A’PB with microparticle cargo hinders tumor suppression. (**A**) Antibody-engineered macrophages/monocytes pre-loaded with cargo prior to treatment. (i) Schematic of in vivo marrow pre-load therapy to assess off-target effects on phagocytosis. Freshly harvested marrow cells phagocytose IgG-opsonized polystyrene microparticles for 1 h prior to tail-vein injections. (ii) Representative images of marrow cells with polystyrene microparticles, with excess particles removed by centrifugation prior to injection. (iii) Quantification of microparticles engulfed per marrow cell in a field of view (FOV). About 40% of marrow cells engulfed up to ~10 particles. (**B**) In vivo phagocytosis of WT B16F10 cells in A’PB marrow treatments. (i) Lung tumor burdens in control (untreated) and 2× A’PB + microparticle pre-loaded groups. Mice were treated with antibody-engineered marrow on days 4 and 6 in addition to tail-vein injections of 250 µg anti-Tyrp1 per Figure 2B through day 13. Mean and s.e.m. (n.s. not significant, Ordinary one-way ANOVA with Dunnett’s multiple comparisons, *n* = 5 per three groups, including 2× A’PB in Figure 5B). (ii) Projected survival curves.

We hypothesized that pre-loading the A’PB macrophages with micron-sized particles will impact their tumor access and/or activity, further modulating their phagocytic ability. Engulfment of polystyrene particles reportedly enhances subsequent phagocytosis of particles and bacteria in vivo [35], but pre-engulfment might also undermine motility including anti-cancer phagocytic activity. [29] To test this possibility, A’PB cells were incubated with IgG-opsonized polystyrene microparticles, with ~40% of cells associated with ~1–10 microparticles (Figure 7A). Microparticle pre-loaded A’PB preparations were injected at day 4 (per Figure 5C) and at day 6 per the ‘2× A’PB’ dosing therapy (plus anti-Tyrp1 per Figure 5B) in the B16F10 lung metastasis model. Mice sacrificed on day 14 showed that the 2× A’PB + microparticles had no significant effect on the tumor area relative to untreated controls (Figure 7B). The result nonetheless shows that A’PB activity can be effectively modulated.

## 3. Discussion

**Figure 8:**
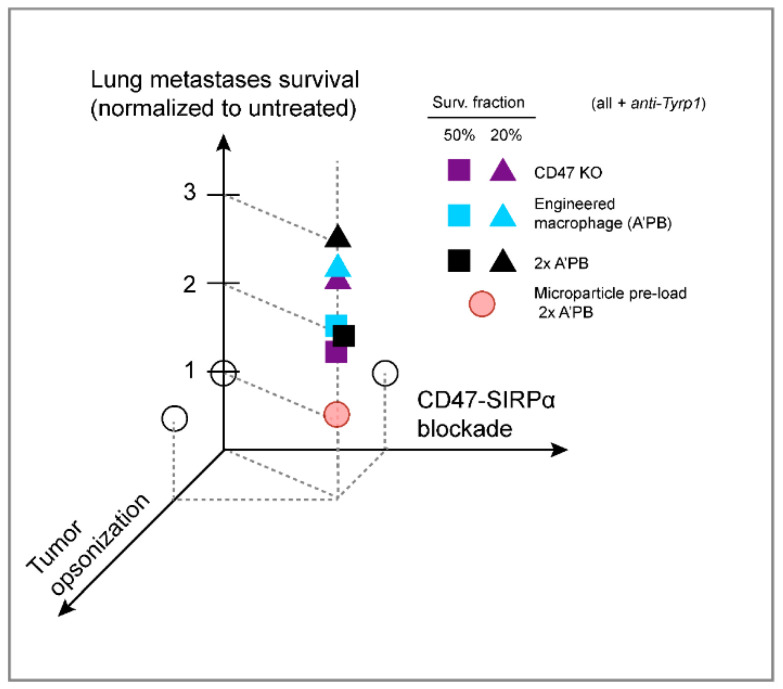
Summary of lung metastases results shows need for combination therapy. Schematic of survival results, with CD47-SIRPα disruption and tumor opsonization extending survival in combination, but not separately based on statistical significance. Each point is representative of median (square: 50% survival) or 80th percentile (triangle: 20% survival) survival time for the indicated group and is normalized to the survival of the untreated.

Here, we show that CD47 ablation is insufficient on its own to affect tumor progression in a poorly immunogenic, metastatic model that is widely used as a pre-clinical model. Our KO and WT control tumors across various B16F10 lung metastases indicate a consistent median survival of ~20–25 days that reflects exponential-like growth (Figure 4A,B for CD47 KO and Figure 5C and Figure 7B for WT B16). Clinical trials also show that CD47 monotherapy is rarely, if ever, effective against human cancer [4,5]. Anti-CD47 blockade in combination with a second opsonizing mAb can show efficacy in the clinic against liquid cancer, but solid tumors likely present larger barriers and limits to anti-CD47-based blockade even in combination treatments. Similarly, subcutaneous B16F10 tumors treated with anti-Tyrp1 show little to no effect of anti-CD47 [24,25]. Our CD47 knockout is 100% (rather than incomplete per earlier studies), and a combination with the tumor-opsonizing IgG TA99 proves effective against established lung metastases of B16F10s. The effect is consistent and significant, with survival extended 2–3-fold across several different studies (Figure 8). Our anti-SIRPα blocked macrophages also proved to be effective in combination with tumor opsonization.

Heterogeneity of lung tumor nodules and explosive growth can be difficult to suppress. Anti-Tyrp1 therapy of mice bearing CD47-deleted metastases nonetheless resulted in lower overall tumor burden even though mice succumbed with similar clinical symptoms as untreated controls. These observations point to a small number of escaping tumors causing moribundity, but otherwise exceptional tumor control by tissue-resident macrophages [36]. The model of phagocytic suppression of rapidly growing B16F10 metastases is extended to slow-growing A549 liver metastases that are effectively repressed by treatment. The A549/NSG model lacks other effector immune cells, consistent with a key role for macrophages and phagocytic function. Overall, our analyses of tumor growth, variability, and phagocytosis provide insight into immune cell-tumor relationships that might translate to a patient’s response to such therapy.

Our results with a standard pre-clinical cancer model contrast with many early reports that anti-CD47 monotherapy can suppress tumor growth in various mouse models (often xenogeneic in immunodeficient mice). The only “monotherapy” to succeed to date against patient tumors is an intratumorally administered SIRPα-IgG1 chimera in fungoides mycosis (a type of lymphoma), with evidence that the IgG1 serves to opsonize while SIRPα binds CD47 [37,38]. However, even low levels of CD47 can block phagocytosis [19,39], and recent studies with subcutaneous tumors of B16F10s engineered to locally secrete anti-CD47 nanobody failed to achieve 100% blockade and extended survival only modestly even with anti-Tyrp1 [25]. The combination of complete CD47 disruption and tumor opsonization is thus not only synergistic but *required* for tumor control.

Our antibody-engineered marrow macrophage/monocyte strategy effectively suppresses growth in two solid tumor models of metastases, and it motivates further quantitative understanding of macrophage infiltration and activity. SIRPα blockade is restricted in our approach to myeloid cells, unlike the blockade of CD47 on all healthy ‘self’ cells. Additionally, a complex interplay of cell biophysics (e.g., membrane fluidity and cellular compliance [35]) and catabolism-metabolism is suggested by our finding that phagocytosed micro-particles modulate the in vivo efficacy of antibody-engineered marrow macrophages/monocytes. We did find that pre-feeding bone marrow-derived macrophages in vitro with red blood cells could inhibit subsequent B16F10 eating, but the in vivo consequences need more study (Figure S9). Pre-loading with fewer, smaller, and/or degradable particles, including viruses, might usefully enhance macrophage phenotype.

## 4. Materials and Methods

### 4.1. Mice

Six- to 14-week-old C57BL/6J mice were procured from The Jackson Laboratory (Bar Harbor, ME, USA). Six- to 10-week-old NOD-*scid* IL2Rγ^nul^ (NSG) mice were procured from the Stem Cell and Xenograft Core at the University of Pennsylvania. To induce lung tumor metastases, 2 × 10^5^ wild-type (WT) or CD47 KO B16F10 cells were injected intravenously in 100 µL of sterile PBS. Initial studies with anti-CD47 were similar but subcutaneous. To induce liver metastases, A549 cells were surgically implanted in the livers of NSG mice and monitored with fluorescence imaging (IVIS Lumina). Donor bone marrow for marrow treatments was isolated from age-matched C57BL/6 or NSG mice using well-established protocols (see Section 4.4). All animal studies were performed according to approved Institutional Animal Care and Use Committee (IACUC) protocols at the University of Pennsylvania.

### 4.2. Quantification of Tumor Burden

Mouse lungs were excised and dissected into five individual murine lung lobes. Harvested lobes were imaged with Nikon D7500 or D3000 DSLR cameras on an SMZ1500 dissecting microscope with an AmScope camera adapter against a 5 × 5 mm^2^ printed grid. Images were loaded into ImageJ and a selection was drawn around each lung lobe. Red, green, and blue color channels were split to reduce false signal from blood, bruising, and other tissue discoloration, and only the red channel was used for quantification. The image was then thresholded with the auto-thresholding algorithm ‘MaxEntropy’. Relative contribution of white pixels (putative B16F10 nodules) and black pixels (normal lung tissue) was calculated for an estimate of total lung tumor area. The 5 × 5 mm^2^ grid enabled conversion from pixel sensor to absolute area. Nodules were counted in experiments with pre-determined sacrifice time points.

### 4.3. Quantitative Analyses for Estimation of Tumor Growth and Projected Survival

To predict long-term survival from single timepoint measurements, nonlinear models (exponential, Area = A_0_ * e^k^*^t^; power law Area = A_0_ * t^B^) were fit to multiple timepoint data (Figure 1Biii,iv) to choose respective growth exponent values for WT and CD47 KO B16F10 tumors. The measured tumor burden at sacrifice for each individual mouse was then fit with an exponential with the determined exponent value. A_0_ for each individual mouse was allowed to vary for the best fit, reflecting individual engraftment environments. The critical survival tumor area threshold of 300 mm^2^ was then used to calculate projected survival time (t, in days) for each mouse with the calculated individual A_0_ and B parameters. Inset plots show representative estimated growth curves for three individual mice within each experiment using this method.

### 4.4. Marrow Harvest and Engineering

Donor mice were sacrificed by CO_2_ inhalation and subsequent confirmatory cervical dislocation. Hind legs were gently dislocated before excision of surrounding fat, muscle, and skin. Femurs and tibiae were separated, cut open at the ends, and centrifuged for up to 25 s at 12,000× *g* to remove marrow from the bones. Pellets were then re-suspended in 1 mL ACK Lysing Buffer for 12 min before centrifugation for 5 min at 300× *g* to aspirate lysed RBCs. Marrow cells were counted with a hemocytometer, adjusted to 8 × 10^7^ cells/mL (2 × 10^7^ cells/250 µL cell injection), and incubated with anti-SIRPα blocking antibody (P84, 4.5 µg/2 × 10^7^ marrow cell injection) at RT for 45 min. Cells were then centrifuged for 5 min at 300× *g* to remove excess anti-SIRPα, then re-suspended again at 8 × 10^7^ cells/mL in 2% fetal bovine serum (FBS) and 1 × 10^3^ µg/mL TA99. For microparticle-loaded experiments, 2.1-µm streptavidin-coated polystyrene microparticles (#SVP-20-5, 0.5% *w*/*v*; Spherotech, Lake Forest, IL, USA) were centrifuged at 16,300× *g* for 20 min and re-suspended in 5% FBS/PBS containing 5 µg/mL anti-streptavidin ( #410501, clone 3.A20.2; BioLegend, San Diego, CA, USA). Particles were then incubated on rotator for 15 min at RT, then centrifuged again 16,300× *g* for 10 min. Marrow cells after the removal of anti-SIRPα (concentration: 8 × 10^7^ cells/mL) were then incubated with the opsonized microparticles for 1 h on rotator at RT in 5% FBS prior to being washed twice with 5% FBS/PBS at 300× *g* for 5 min and re-suspended in the final cell solution as above. A total of 250 µL of these cell solutions were then administered intravenously to strain-matched mice bearing tumors. A second cell dose in “2× A’PB” conditions was administered i.v. on day 6 post inoculation. A total of 600 µg doses of anti-human (Rockland #209-4139) were similarly injected to opsonize in A549 tumor experiments instead of TA99.

### 4.5. Cell Culture

B16F10 melanoma cells were obtained from ATCC (Manassas, VA, USA). Cells were cultured at low passage number in RPMI-1640 until at least one passage before a planned tumor inoculation or phagocytosis assay, when the media was switched to DMEM. B16F10 cells melanize and exhibit slower growth rates in DMEM compared to RPMI. Isolated primary bone marrow cells are differentiated for 7 days in Iscove’s Modified Dulbecco’s Medium (IMDM) supplemented with 10% (vol/vol) FBS, 1% penicillin-streptomycin (P/S), and 20 ng/mL M-CSF to produce bone-marrow derived macrophages (BMDMs) for in vitro assays (BioLegend). A549 lung carcinoma cells were cultured in F-12 media, supplemented as above. For B16F10 tumor disaggregation, mouse lungs were excised and disaggregated with dispase ( #354235; Corning, NY USA) supplemented with 3 mg/mL collagenase and DNaseI prior to cell culture in DMEM.

### 4.6. CRISPR-Cas9 Deletion of CD47

CRISPR/Cas9-mediated knockouts of *Cd47* were generated using LentiV_Cas9_puro (Addgene #108100) and Lenti_sgRNA_EFS_GFP plasmids (Addgene #65656), which were gifts from Christopher Vakoc. The following single guide RNA (sgRNA) oligonucleotides was used: *Cd47*, 5′-TCCCCGTAGAGATTACAATG. All sgRNA oligonucleotides were designed using the Broad Institute’s GPP sgRNA Design tool and purchased from Integrated DNA Technologies. sgRNA targeting sequences were annealed and ligated into the Lenti_sgRNA_EFS_GFP expression vector with Esp3I (Thermo Fisher Scientific, Waltham, MA, USA).

Lentivirus was generated in individual wells of a 6-well plate (Corning) by transfecting HEK293T cells with 165 ng pVSVg, 1.35 ug psPAX2, and 1.5 ug lentiviral transfer plasmid using 7.5 ul TransIT-LT1 Transfection Reagent (Mirus Bio, Madison, WI, USA) per well following the manufacturer’s protocol. Viral supernatants were collected 48 h after transfection, centrifuged at 300× *g* for 5 min to remove cell debris, and then added to targets cells at a 1:1 volumetric ratio of viral supernatant to culture media. To generate a stable Cas9-expressing B16F10 line, cells successfully transduced with LentiV_Cas9_puro were selected using puromycin (Invitrogen, Waltham, MA, USA) at 2 ug/mL for one week. Stable Cas9-expressing B16F10 cells were then again transduced with lentivirus for sgRNA expression vectors with targeting sequences against *Cd47*. Individual clones were generated by serial dilution and selecting only GFP-positive clones. Clonal knockout was confirmed by flow cytometry using anti-mouse CD47 (clone MIAP301).

### 4.7. Phagocytosis Assays

C57BL/6J bone marrow was harvested as described above, plated in 100 mm untreated Petri dishes in IMDM (supplemented 10% FBS, 1%P/S, 20 ng/mL M-CSF), and allowed to differentiate into macrophages for 7 days. On day 7 of differentiation, macrophages were washed with 1x PBS, lifted with 0.25% Trypsin for 10 min at 37 °C, and plated in 24- or 6-well plates in the same supplemented IMDM for phagocytosis assays the following day. Targets for phagocytosis were opsonized for up to 30 min at RT or on ice at the following concentrations and antibodies: 10 µg/mL TA99 (BioXCell, Lebanon, NH, USA) for B16F10 variants, and 10 µg/mL anti-streptavidin for polystyrene microparticles. Where applicable against WT targets, mouse targets were CD47-blocked with 10 µg/mL MIAP301 (BioXCell). Prior to adding targets to the wells for phagocytosis, macrophages were labeled with 0.5 µM CellTracker DeepRed in PBS for 15 min and B16F10 cells with 10 µM Vybrant CFDA SE Cell Tracer Kit in PBS for 15 min at RT. Wells were washed with serum-free IMDM (IMDM supplemented with 0.1% BSA, 1% P/S, no M-CSF or FBS) after 2 h. For imaging, wells were fixed for 20 min in 4% paraformaldehyde at RT, then washed with PBS, and stored at 4 °C in PBS prior to imaging.

### 4.8. Immunohistochemistry

Lungs harvested from mice were fixed in 4% paraformaldehyde overnight at 4 °C and stored in 70% ethanol. Lungs embedded in paraffin and sectioned by the Molecular Pathology and Imaging Core or the Comparative Pathology Core at the University of Pennsylvania. Staining for immune markers followed established core protocols.

### 4.9. Statistical Analyses and Equation Fitting

Results were analyzed for statistical significance, and equations were fit both with built-in analyses in Prism 9.0.1.

## 5. Conclusions

CD47 is a promising, emerging target for clinical cancer therapeutics but its efficacy remains a challenge. Pro-phagocytic signals are required in combination with deep disruption of CD47 inhibitory signaling. The clinical success of CD47-based therapeutics depends on macrophage activation, not just inhibition of “don’t eat me” signals. We demonstrate this strategy in two different models of metastasis by CD47 deletion and by adoptive transfer of SIRPα-blocked marrow cells in combination with tumor-opsonizing IgG. While advancing our understanding of phagocytosis and CD47′s role in solid tumors, further efforts might seek to: (i) maximize CD47-SIRPα disruption, (ii) increase the numbers of phagocytic effector cells [40], and (iii) provide additional pro-phagocytic signals (e.g., another tumor-specific monoclonal antibody, bispecific antibodies, some chemotherapeutics, etc.) that are suppressed by CD47-SIRPα.

## Data Availability

Data are openly available at https://doi.org/10.6084/m9.figshare.19358786 (accessed on 30 January 2022).

## Supplementary Materials

The following supporting information can be downloaded at: https://www.mdpi.com/article/10.3390/cancers14081930/s1. Figure S1: Cell surface marker expression levels in B16F10 lines. Figure S2: Wild-type B16F10 metastases do not respond to low or high doses of anti-Tyrp1. Figure S3: Combination anti-CD47 and anti-Tyrp1 fails to affect tumor area of primary wild-type B16F10 tumors. Figure S4: Computed tomography (CT) imaging of B16F10 lung metastases (CD47 KO). Figure S5: Melanin and nuclei of tumor and lung tissue in embedded lung sections. Figure S6: Marker-free analysis of immune infiltrate in treated CD47 KO lung tumor nodules. Figure S7: Tumor masses on lungs (related to Main Figure 2). Figure S8: Tumor growth in A’PB-treated mice. Figure S9: Pre-feeding bone marrow-derived macrophages with non-tumor targets can affect on-target tumor cell phagocytosis.
