# Supplementary material for: CD47-SIRPα Checkpoint Disruption in Metastases Requires Tumor-Targeting Antibody for Molecular and Engineered Macrophage Therapies
Source: Cancers (Basel). 2022 Apr 11;14(8):1930. doi: 10.3390/cancers14081930 (PMC9026896; doi:10.3390/cancers14081930)
Supplement: Supplementary file 1 [file cancers-14-01930-s001.zip › cancers-1609218-supplementary.pdf]

**Supplemental Figure Legends**

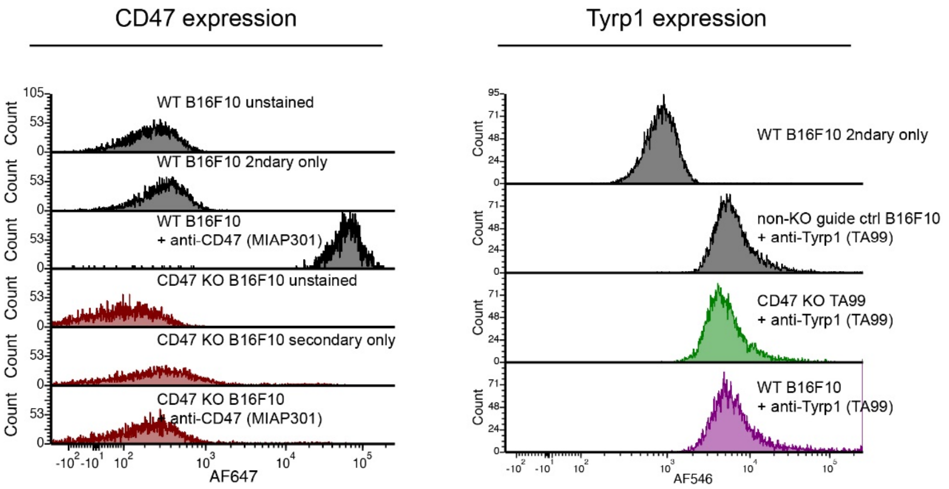


**Figure S1. Cell surface marker expression levels in B16F10 lines**.

**A** CD47 surface expression is completely abolished via CRISPR/Cas9 editing. Anti-CD47 (clone: MIAP301) was used as the primary antibody and AlexaFluor647 as the secondary antibody prior to measurement by flow cytometry.

**B** Tyrp1 is expressed on the surface of the B16F10 cell surface and is thus a target for IgG opsonization and Fc receptor-mediated phagocytosis. Anti-Tyrp1 (clone: TA99) was used as the primary antibody and AlexaFluor546 as the secondary antibody prior to measurement by flow cytometry.

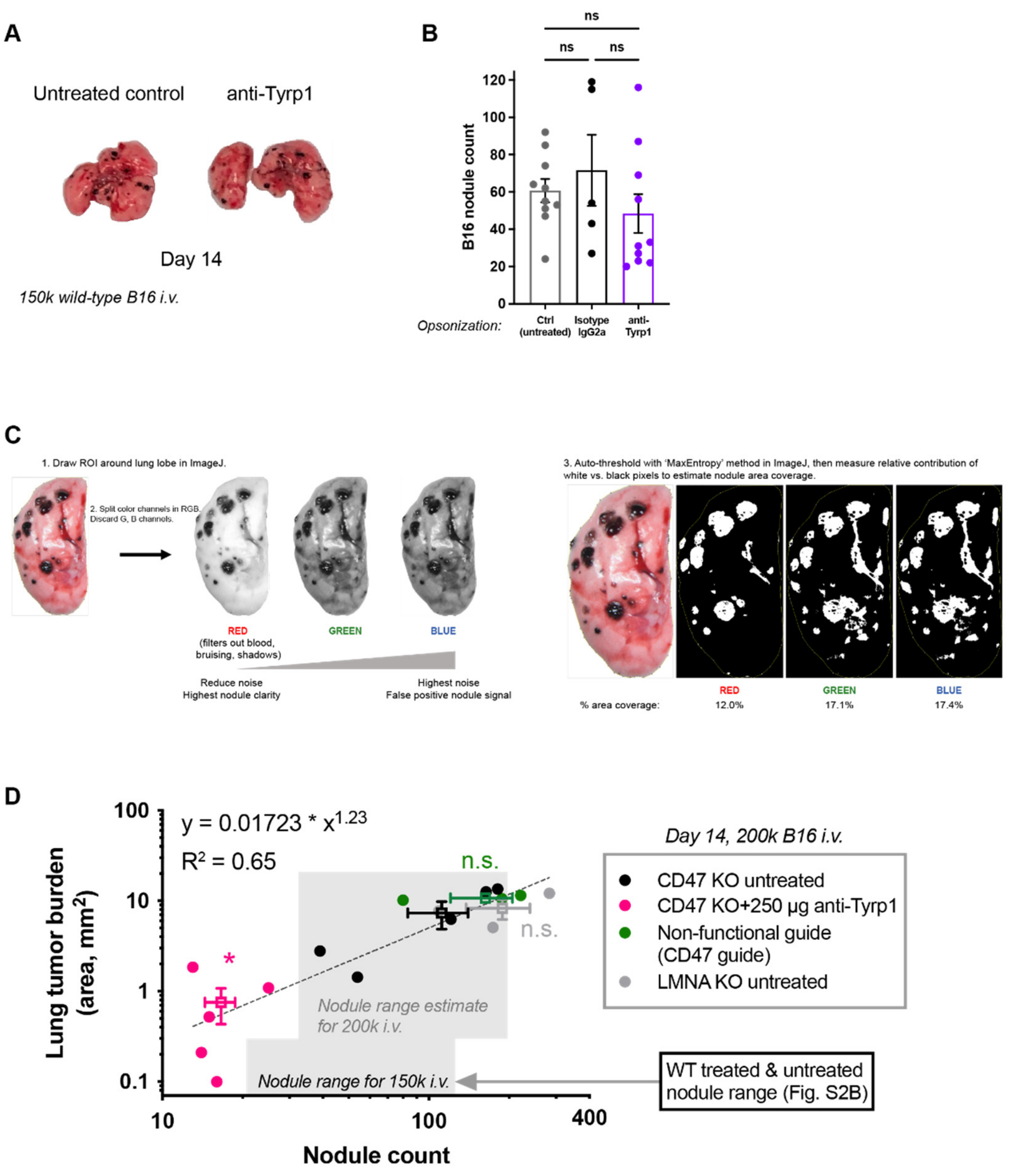


**Figure S2. Wild-type B16F10 metastases do not respond to low and high doses of anti-Tyrp1.**

**A** Representative micrographs of lungs from mice bearing metastases at day 14 post inoculation of 1.5 x $10^5$ wild-type B16F10s i.v. Tumors are allowed to establish, and mice are then intravenously injected with 250 µg of anti-Tyrp1 intravenously on days 4, 5, 7, 9, 11, and 13 post inoculation. Mice are sacrificed and organs are surveyed for B16F10 metastases. Same statistical analyses as Fig.1A-i.

**B** Quantification of tumor burden. WT B16F10 nodules were counted manually. No significant differences were detected at day 14 (see Figure 1A and 4B for 2 x $10^5$ WT B16F10s i.v.). Results shown are from 3 independent experiments (n = 5 per group within each experiment, open circles indicate results from groups in concurrent experiments, gray symbols indicate isotype IgG2a control treated (250 µg i.v. on the same timeline)), black symbols indicate untreated/non-injected, mean ± s.e.m., n.s. p > 0.05, Ordinary one-way ANOVA with Sidak's multiple comparisons).

**C** Workflow for quantification of tumor burden on lung lobe surfaces. B16F10 tumor burden was quantified in FIJI by analyzing black/melanized tumor area relative to normal lung tissue area. Excised lungs were imaged with a dissecting microscope and a DLSR. ROIs were drawn around each lung lobe and color channels were split in to RGB. The R channel shows the highest signal-to-noise for tumor area. To reduce bias from manual thresholding, all images were auto-thresholded with the 'MaxEntropy' method in FIJI, then the relative contribution of white (tumor) pixels and black (normal lung tissue) pixels was measured.

**D** Scaling relationship between nodule count and tumor area on day 14 as estimates of tumor burden (2 x $10^5$ i.v. cell inoculation number for all groups). Different B16F10 variants are shown, including a wild-type CD47-expressing variant, CD47 KO, CD47 KO plus anti-Tyrp1 (TA99), and a nuclear lamin (LMNA) KO clone. Each solid point represents lung tumor burden on one mouse, and each open point represents the average value (mean ± s.e.m., n.s. p > 0.05, Ordinary one-way ANOVA with Sidak's multiple comparisons, indicated statistical comparison is for the x parameter relative to CD47 KO). Gray shaded boxes show estimate of regimes for 1.5 x $10^5$ and 2 x $10^5$ i.v. cell inoculation numbers, respectively as indicated, on day 14 post inoculation.

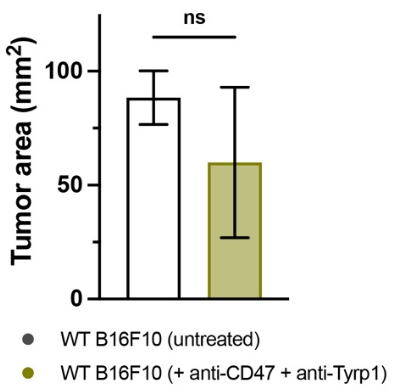


**Figure S3. Combination anti-CD47 and anti-Tyrp1 fails to affect tumor area of primary wild-type B16F10 tumors.**

Tumor area comparing untreated and combination treated with anti-CD47 anti-Tyrp1 i.v. on Days 4, 5, 7, 8, 11, 13, and 15 post inoculation of 2 x $10^5$ cell WT B16F10 cells [41]. Anti-CD47 + anti-Tyrp1 fails to affect tumor area on day 15 relative to untreated controls (n = 6 per group, n.s. $p > 0.05$ Welch's t-test with Welch's correction).

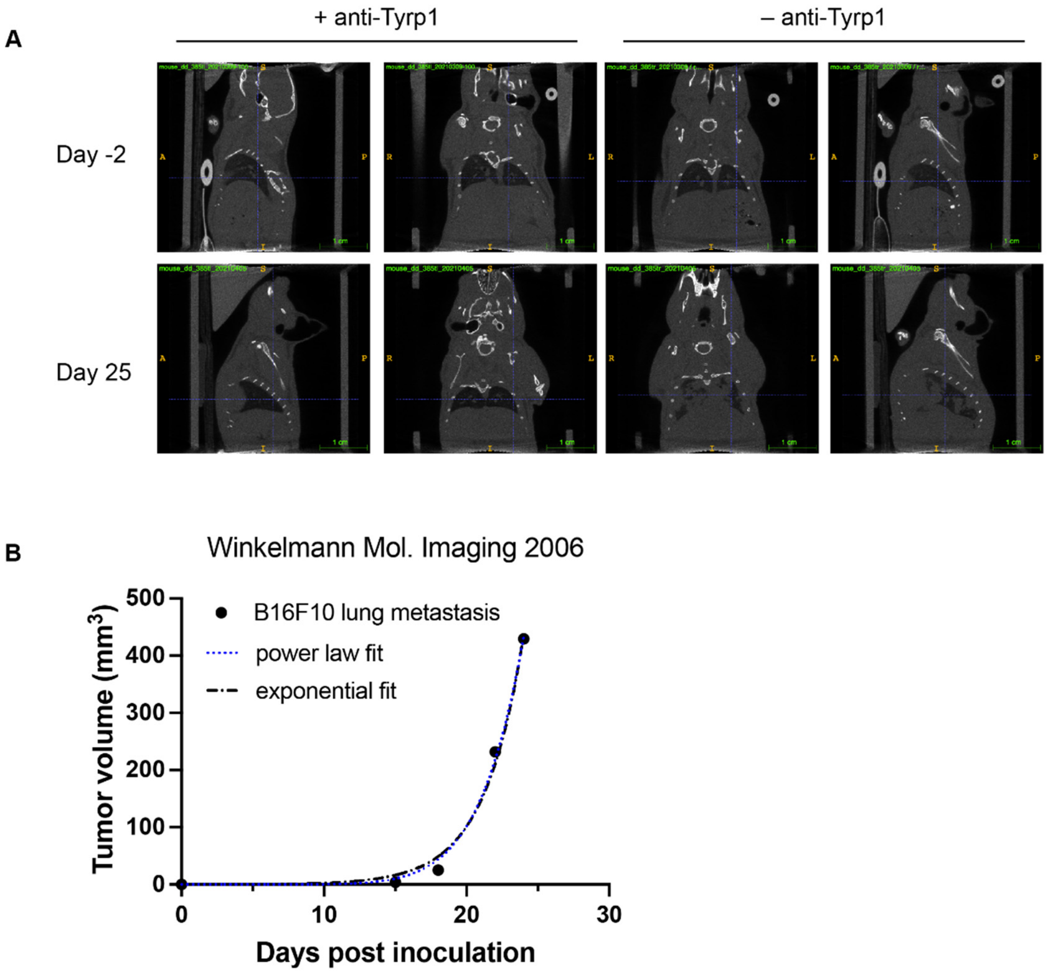


**Figure S4. Computed tomography (CT) imaging of B16F10 lung metastases (CD47 KO).**
**A** Representative microCT images of mice bearing CD47 KO lung metastases on day –2 and day 25 post inoculation. Mice were inoculated with tumor cells and treated with anti-Tyrp1 or left untreated as described in Figure 1Bi. Anti-Tyrp1-treated mice (left) exhibit low tumor burden at Day 25 relative to untreated (right) mice.
**B** Power law and exponential fits to previously reported microCT data of B16 lung metastases [27].

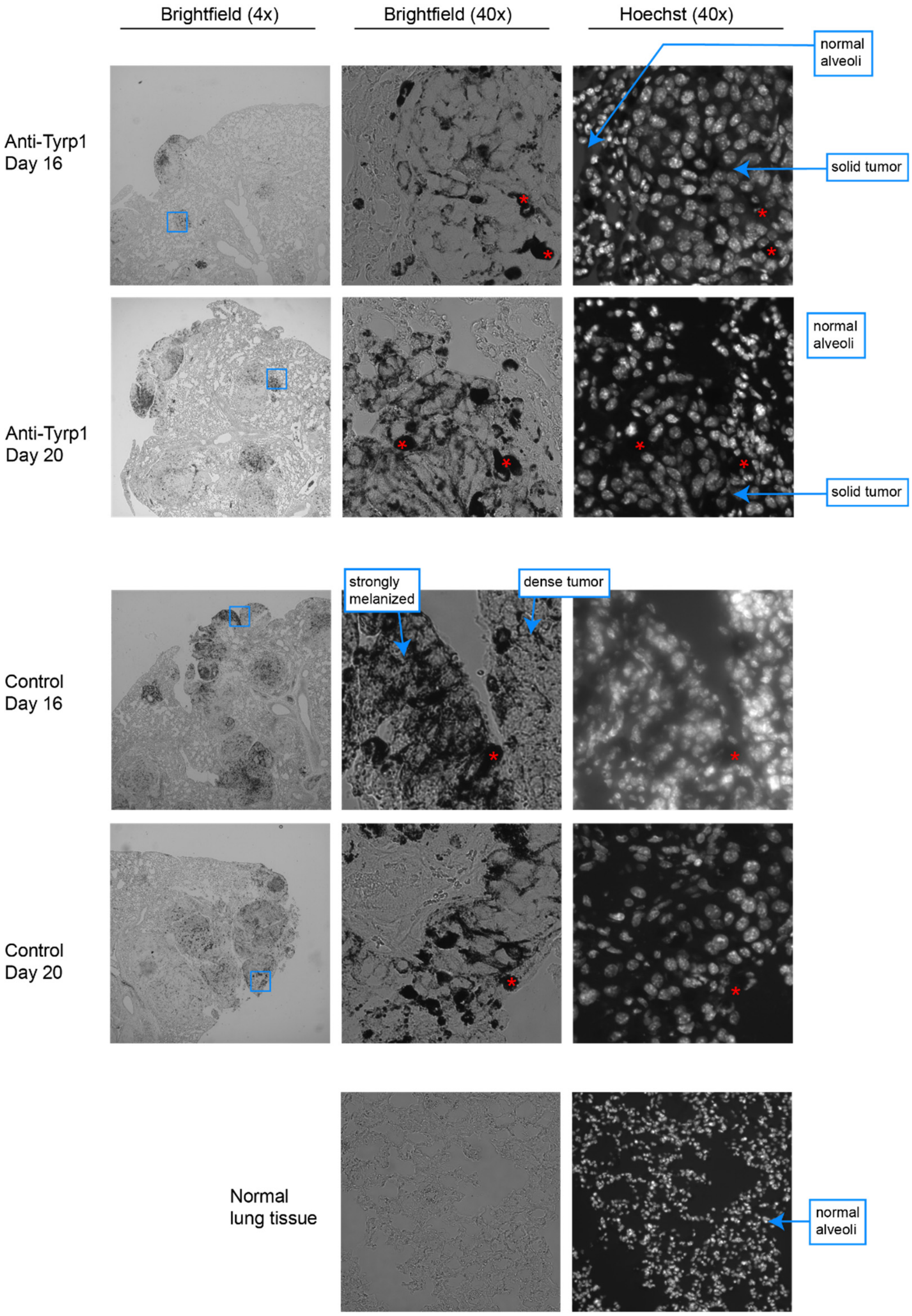


**Figure S5. Melanin and nuclei of tumor and lung tissue in embedded lung sections.**

*Top panel*: Brightfield images (4x and 40x) and DNA-stained/UV channel images (40x) of anti-Tyrp1-treated lungs. Tumor nodules are present and disturb the surrounding tissue as dense, solid masses co-localized with melanin (black) granule deposition. B16F10 nuclei are large and densely packed relative to small, bright nuclei of the surrounding normal tissue.
*Bottom panel:* Brightfield images (4x and 40x) and DNA-stained/UV channel images (40x) of control (untreated) lungs. B16F10 tumors are widespread in size and melanin deposition. Normal alveoli in the absence of tumor are shown in comparison.

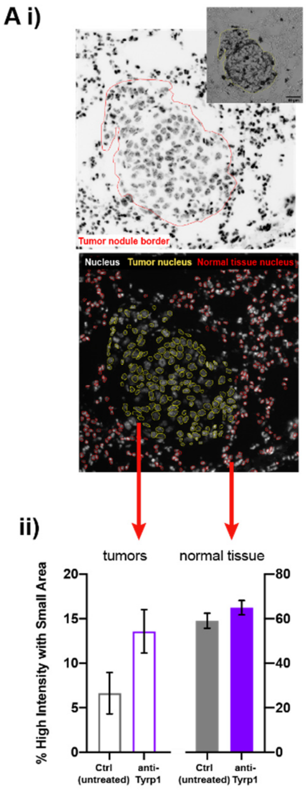


**Figure S6. Marker-free analysis of immune infiltrate in treated CD47 KO lung tumor nodules.**

**A (i)** Inverted contrast of DAPI-stained nuclei in a nodule (red outline) or brightfield (yellow), with nuclear outlines to quantify size and intensity in tumors and adjacent tissue. **(ii)** Fractions of normal nuclei with high intensity and small areas in tumors and adjacent tissues (mean ± s.e.m.)

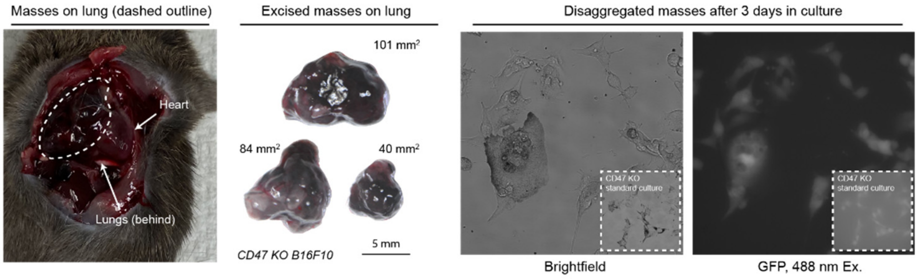


**Figure S7. Tumor masses on lungs (related to Main Figure 2).**
Representative images of dissected mice with large tumor (CD47 KO B16F10) masses compressing the lungs and chest cavity, despite reduced tumor burden on the lungs. These masses were disaggregated with Dispase supplemented with collagenase and DNaseI and plated for 3 days in DMEM to confirm their identity as B16F10 tumor cells (GFP+, 488 nm Ex.).

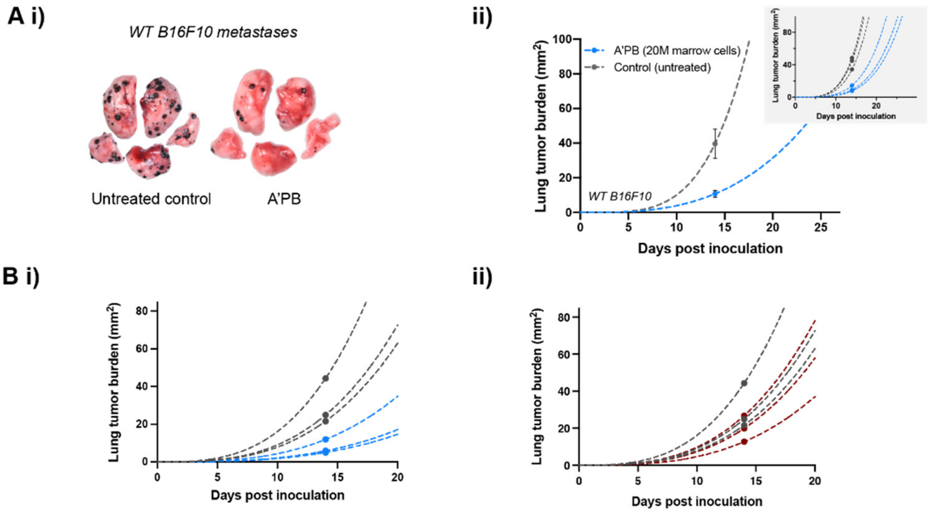


**Figure S8. Tumor growth in A'PB-treated mice.**

**A (i)** Lung tumor burden as assessed on day 14 post inoculation. Representative photographs of lung tumor nodules in A'PB-treated lungs and untreated control lungs.

**(ii)** Power law (Area = $A_0 * t^B$) fits growth ($A_{0,ctrl}$ = 0.00088, $A_{0,A'PB}$ = 0.0039, $B_{ctrl}$ = 4.1, $B_{A'PB}$ = 3.0). Inset: projected power law growth curves of representative individual control and 1xA'PB-treated mice (n = 3 each).

**B (i)** Projected power law growth curves of representative individual control and 2xA'PB-treated mice (n = 3 each).

**(ii)** Projected power law growth curves of representative individual control and microparticle-preloaded 2xA'PB-treated mice (n = 3 each).

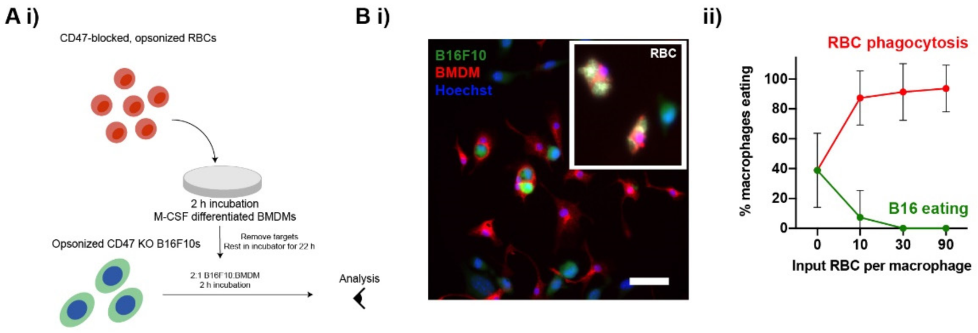


**Figure S9. Pre-feeding bone marrow-derived macrophages with non-tumor targets can affect on-target tumor cell phagocytosis.**

**A** Schematic of *in vitro* pre-feed phagocytosis assay to assess off-target effects on phagocytosis. Macrophages are allowed to phagocytose IgG-opsonized, CD47-blocked human/mouse red blood cells (RBCs) 24 h prior to phagocytosis of TA99-opsonized, CD47-deleted B16F10s. Bone marrow-derived macrophages are plated for 7 days in IMDM supplemented with 20 ng/mL M-CSF prior to assay start.

**B (i)** Representative fluorescence image (40x magnification) of macrophage engulfment of CD467 KO B16F10s in phagocytosis assays. Macrophages engulf B16s without pre-feed (main image), while RBC-pre-fed macrophages fail to phagocytose target B16s (inset). **(ii)** Quantification of fraction of phagocytic macrophages pre-fed with either RBCs or microparticles. Macrophages engulf anti-Tyrp1-opsonized CD47 KO B16F10s readily without pre-feeding (0:1 ratio input target:macrophage). High RBC phagocytosis blocked B16F10 phagocytosis completely.
